# Research on an intelligent diagnosis method of mechanical faults for small sample data sets

**DOI:** 10.1038/s41598-022-26316-6

**Published:** 2022-12-20

**Authors:** Jun Zhao, Yuhua Shi, Feng Tan, Xufeng Wang, Youqiang Zhang, Jiean Liao, Fan Yang, Zhanhong Guo

**Affiliations:** 1 College of Mechanical and Electrical Engineering, Tarim University, Aral, China; 2 Chongqing University of Posts and Telecommunications, Chongqing, China

**Keywords:** Mechanical engineering, Computational science

## Abstract

The difficulty of feature extraction and the small sample size are two challenges in the field of mechanical fault diagnosis for a long time. Here we propose an intelligent mechanical fault diagnosis method for scenario with small sample datasets. This method can not only diagnose bearing faults but also gear faults, and has strong generalization performance. We use convolutional neural network to realize automatic feature extraction. Through sliding window scanning, one sample set is expanded to three sub-sample sets with different scales to meet the needs of deep learning training. Three convolutional networks are used to extract the features of the subsets respectively to ensure that their useful features are fully extracted. After feature extraction, the feature is reconstructed through feature splicing. Because of the unique advantages of SVM in dealing with small sample sets, we use SVM to classify the reconstructed features. We use the bearing data set collected by Case Western Reserve University in the United States, the bearing fault data set collected by Xi'an Jiaotong University in China, and the gearbox fault data collected by the University of Connecticut in the United States to conduct experiments. The experimental results show that the accuracy of training, validation and testing of the proposed method on the three data sets all reach 100%. This proves that our method can not only tackle the two challenges, but also has high fault diagnosis accuracy and strong generalization performance. It is hoped that our proposed method can contribute to the development of mechanical fault diagnosis.

## Introduction

Since the Industrial Revolution in the 1860s, machinery has been related to all aspects of our lives, from national strategic equipment to daily travelling vehicles. Due to the long-term service, complex working conditions and harsh working environment of mechanical equipment, its parts are easy to be damaged during operation, which may lead to component failure^1,2^. In serious cases, it may even cause major safety accidents and economic losses^3,4^. Therefore, the health status monitoring and fault diagnosis of mechanical systems are of great significance in ensuring the safety and reliability of mechanical systems^5–7^.

In the working process of mechanical equipment, its vibration response often contains rich information of equipment health status. Mechanical vibration has the advantages of simple measurement and convenient analysis properties. Therefore, the use of mechanical vibration signals for health monitoring and fault diagnosis of mechanical equipment has received extensive attention from scholars, researchers, college teachers, etc^8^. Bearing and gear, as important parts of mechanical equipment, are especially prone to failure in the working process. They have attracted many attentions to study their fault diagnosis^9–16^. Most of them relies on professional knowledge and experience to decompose the vibration signal into multiple sub signals through certain signal processing techniques, and then extract the useful components as features. This kind of feature extraction method requiring professional knowledge and experience is the first challenge for mechanical fault diagnosis.

In 2006, Hinton^17^ and other scholars put forward the concept of deep learning, which makes AI become a research hotspot again, and is widely used in image processing^18^, speech recognition^19^, natural language processing^20^ and other fields. As a typical network in deep learning, CNN is widely used in image processing, such as the well-known Google Net, Alex Net, VGG and other networks^21,22^. CNN was originally proposed for image processing (2D data or 3D data), but due to its excellent feature extraction ability, many scholars have introduced CNN into the field of fault diagnosis. Currently, there are two main methods for fault diagnosis using CNN: The first is to transform one-dimensional vibration signals into two-dimensional images in a certain way, so that they can be processed by CNN^23,24^. The second is to transform the model structure of CNN, such as converting the 2D convolution kernels into 1D convolution kernels, so that they can be directly used for 1D data processing^25,26^. Many scholars have also tried this method, and achieved good diagnostic results. However, no matter which method is used, a large amount of training data is required to train the network parameters, and it is difficult to obtain sufficient and balanced sample data^27^ in actual industrial systems. Therefore, small dataset size is the second challenge for mechanical fault diagnosis.

Although many scholars have made achievements by using CNN, most of the studies are conducted on a dataset or a data set, and the research on the generalization performance of the proposed model or method is insufficient. For example, the fault diagnosis effect of the proposed method on the bearing data set is very good, but the effect on the gear data set is not necessarily ideal. To make it generalize, the model structure may need to be partially adjusted, or even the model may need to be rebuilt. Therefore, how to improve the generalization ability of fault diagnosis methods and make them applicable to a variety of domains is also a challenge for mechanical fault diagnosis using CNN.

In order to deal with the above challenges, in this paper, we propose an intelligent diagnosis method for mechanical faults suitable for small sample sets. This method mainly includes two key parts: First, we scan and expand the original sample set through the sliding window to form three new sample subsets with different scales, so that more features can be extracted from the subsets of different dimensions during model training. This improves the fault identification accuracy and generalization performance and can also meet the needs of deep learning training parameters. Second, we design a convolutional neural network structure as a feature extractor to achieve automatic feature extraction. We conducted experiments on three fault data sets (including two bearing fault data sets and one gear fault data set) to verify the effectiveness and generalization performance of the method. The experimental results indicate that the training accuracy, validation accuracy and test accuracy of the proposed method on the three data are all 100%, which proves the effectiveness of the proposed method and its strong generalization performance. The proposed method may also be used for processing other fault data, but it has not been verified by experiments.

The main innovation of our proposed method is the design of a unique feature extractor structure through a conventional one-dimensional traditional neural network (1D-CNN). It is able to automatically extract useful features from the samples. The extracted features are reconstructed by feature stitching. Although the reconstructed feature set is also a small sample set, it can achieve high recognition accuracy even when using a traditional support vector machine (SVM) as a classifier. Therefore, our proposed method can effectively deal with the two long-term challenges in the field of mechanical fault diagnosis.

## Result

### Data preparation

In the experiment, we used three data sets, namely, the bearing data set collected by Case Western Reserve University in the United States, the bearing fault data set collected by Xi'an Jiaotong University in China, and the gearbox fault data set collected by the University of Connecticut in the United States. The data set of Case Western Reserve University in the United States includes four types of data: inner race fault, outer race fault, rolling element fault and normal bearing. Because the dimensions of each type of data collected are inconsistent, we split the samples with long dimensions in half for convenience, and cut other samples with the minimum sample length to make all sample dimensions consistent. We study the data collected by the accelerometer at the driving end. After processing, we finally get a data set with 82 samples, including 24 inner ring faults, 18 outer ring faults, 24 rolling element faults and 16 normal bearings. Each sample contains 120,617 data points. We label the samples with inner ring failure, outer ring failure, rolling element failure and normal bearing with 1, 2, 3 and 4 respectively, and randomly shuffle them. We take 70%, 20% and 10% of the data as the training set, the validation set and the test set respectively.

The fault data in the bearing fault data set of Xi'an Jiaotong University in China are collected under three working conditions. Each working condition includes four types of fault data such as bearing inner and outer ring faults and normal bearing data. There are five types of data in total. We take the data under one of these working conditions for research, that is, the working frequency of the motor is 35 Hz, and the load is 12 KN. During data collection, sensors are arranged in both the vertical and horizontal directions of the bearing end cap, and the data collected in both the horizontal and vertical directions contain bearing fault information. Therefore, we only take the data collected in the vertical direction for experiment. The number of fault samples of each type in the data set is different. For the convenience of research, the number of fault samples of each type is set equal to that of the type with the least number of samples which is 52. After this cleaning, the new dataset contains 260 samples with 52 samples for each type and 32,768 data points of each sample. Similarly, 1–5 is used to label the data, and p–t is used to map and describe the data to show the difference.

The gearbox fault data set of the University of Connecticut in the United States includes 8 types of fault data such as pitting corrosion on the tooth surface, tooth fracture, tooth surface wear, and normal data of the gear, a total of 9 types of data. This data set contains 936 samples with 104 samples for each type and 3600 data points for each sample. We label the data with 1–9 respectively. In order to distinguish it from the data label of Case Western Reserve University, use a-i to correspond with it, so that there will be no confusion in drawing and description. A summary of the characteristics of the 3 datasets is shown in Table 1.

**Table 1:** Dataset characteristics.

Data set	Category label	Number of samples	Sample dimension	Total number of samples
Bearing data set of Case Western Reserve University	1	24	120,617	82
	2	18	120,617	
	3	24	120,617	
	4	16	120,617	
Bearing Data Set of Xi'an Jiaotong University	p	52	32,768	260
	q	52	32,768	
	r	52	32,768	
	s	52	32,768	
	t	52	32,768	
Gear data set of University of Connecticut	a	104	3600	936
	b	104	3600	
	c	104	3600	
	d	104	3600	
	e	104	3600	
	f	104	3600	
	g	104	3600	
	h	104	3600	
	i	104	3600	

### Sample expansion

**Figure 1:**
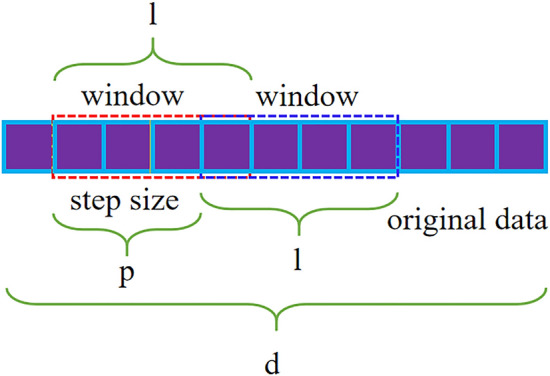
Scanning diagram of sliding window.

Due to the small sample size of the mechanical fault vibration data set, the original data needs to be expanded first to meet the needs of deep learning. To ensure the diversity of samples, we use sliding windows to scan the original data on multiple scales. Professor Zhou^28^ found through experiments that for the original data with d dimension characteristics, when using d/16, d/8 and d/4 windows for scanning, it can not only ensure the diversity of samples, but also maximize the computational efficiency. Therefore, we also use this window size for scanning. The schematic diagram of sliding window scanning is shown in Fig. 1. The dimension of the original data is d, the window size is l, it can be d/16, d/8 or d/4, and the sliding step size is p. Through this sample expansion, we can obtain the sample size needed. Suppose that a single sample can be expanded to N samples, and the value can be calculated by the following formula:

1 $$N = \left[ {\frac{d - l}{{p - m^{\prime}}} + 1} \right]$$

where d is the original sample length; l is the window length; p is the window sliding step size; $m^{\prime}$ is the amount of overlap between adjacent samples; [.] is a downward rounding function.

### Feature extractor design

**Figure 2:**
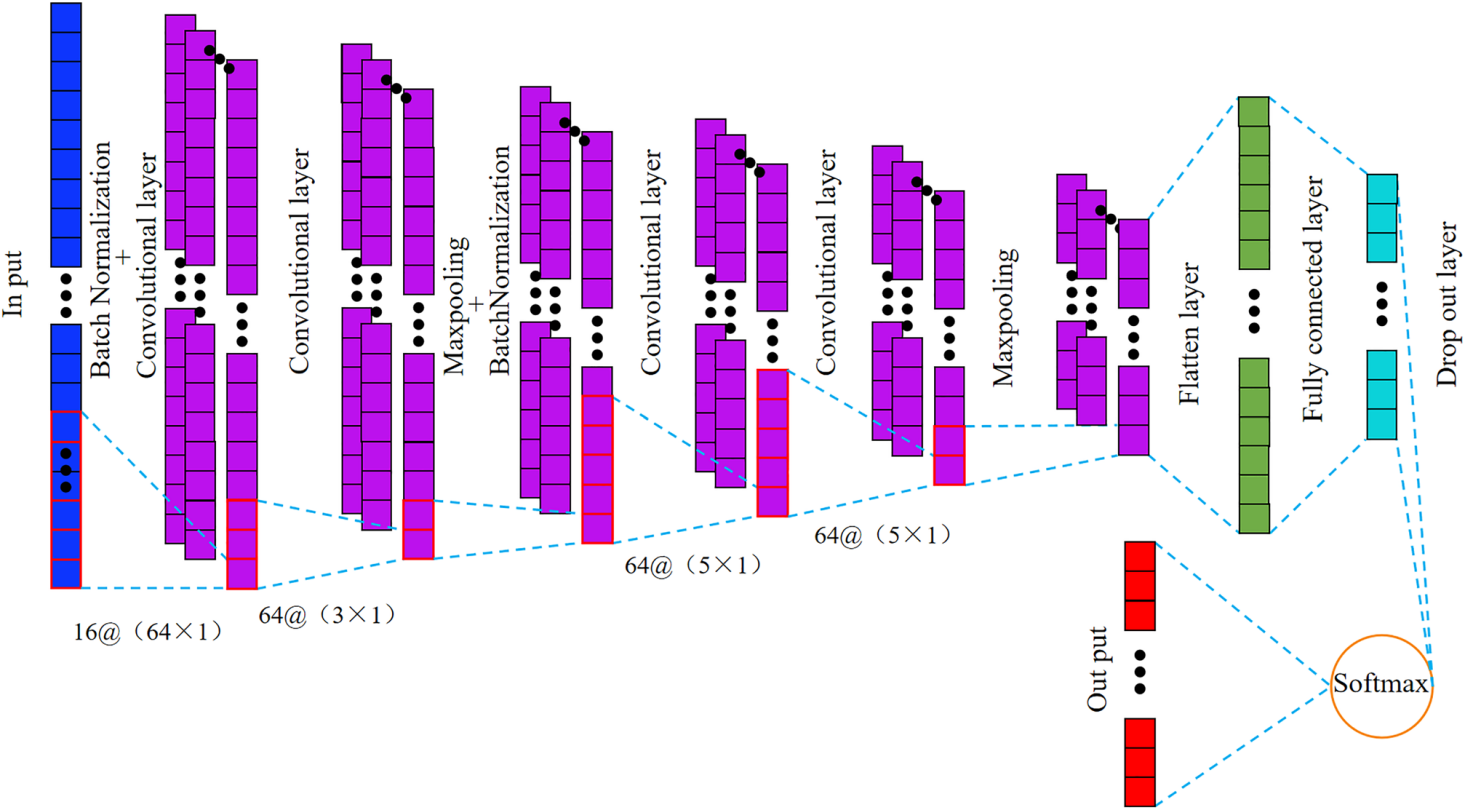
Structure of feature extractor.

The model structure of the feature extractor we designed is shown in Fig. 2. The feature extractor consists of one input layer, four convolution layers, two pooling layers, two batch normalization layers, one flattening layer, one dropout layer, one full connection layer and one softmax layer. Here we designed 4 convolutional layers because we found that the average identification accuracy is about 90% when the number of convolutional layers is 2 or 3, and there are more misclassifications among various types; while the average identification accuracy is already 100% when the number of convolutional layers is 4, so increasing the number of convolutional layers does not improve the identification accuracy and causes a waste of computational resources, so we determined the number of convolutional layers to be 4. The pooling layer is added to reduce the network parameters and computational effort by reducing the dimensionality of the features learned from the convolutional layer. The common pooling layers are maximum pooling and average pooling, and we choose maximum pooling. Usually, a pooling layer is added after the convolutional layer, but considering that the shortest dimension of our dataset is 225, it may lead to the feature dimension of the last pooling layer is less than 1, and there is a similar effect to pooling when the sliding step of the convolutional kernel is larger than 1. Therefore, in order to retain more feature information, we add a pooling layer after every two convolutional layers. The batch normalization layer is similar to data normalization in that it can effectively reduce internal covariate transfer, but our network is not deep, with only 4 convolutional layers, so we only add the batch normalization layer to the first two convolutional layers.

The parameters of each layer are shown in Fig. 2. For example, 16@(64 × 1) represents that 16 dimensions are 64 × 1, the sliding step of the first convolution kernel is set to 1, and the sliding step of the other convolution kernels is set to 2.The first convolutional layer uses a larger convolutional kernel to increase the perceptual field to obtain more data and provide richer information for the subsequent layers. The 2nd, 3rd and 4th convolutional layers use small size convolutional kernels to extract more detailed features. The first convolution kernel has a sliding step of 1 to obtain more information, and the rest is 2 to reduce the feature dimension to improve the network computational efficiency. The flattening layer is added to transform the features extracted from the convolution layer into 1D form to suit for following full connection layer processing. To reduce the over fitting of the network, a dropout layer is added between the full connection layer and the softmax layer. Its function is to inactivate some neurons randomly during the training process to avoid over fitting. The drop rate is set to 0.3. The discard rate is too low to avoid overfitting, and too high to avoid underfitting, so we set a more intermediate value. To further reduce the over fitting of the model, we add a regularization term to the full connection layer. The fully connected layer is the layer from which features are extracted by the feature extractor. If there are too many neurons in the fully connected layer, the dimensionality of feature reconstruction will be too high, and too few neurons in the fully connected layer may lead to the loss of some important features. Therefore, we set the total connective layer neurons to 64, a relatively intermediate value.

The Relu function is often chosen for all convolution layers and full connection layers as activation functions in the model^29^. Because it is sparse, it allows the sparse model to better mine the relevant features to fit the training data. Its mathematical description is shown in Eq. (2).

2 $$\begin{aligned} a_{j}^{h} (i) = f(y_{j}^{h} (i)) = & \max (0,y_{j}^{h} (i)) \\ = & \left\{ \begin{gathered} y_{j}^{h} (i),y_{j}^{h} (i) \ge 0 \hfill \\ 0,y_{j}^{h} (i) < 0 \hfill \\ \end{gathered} \right. \\ \end{aligned}$$

where $a_{j}^{h} (i)$ is the activation value of $y_{j}^{h} (i)$; $y_{j}^{h} (i)$ is the i-th output after the j-th convolution operation in layer h.

The commonly used loss function species are cross-entropy loss function and mean squared error loss function, but the cross-entropy loss function is usually chosen because it can avoid the problem of reduced learning rate of the mean squared error loss function^30^. The mathematical description of the cross-entropy loss function is shown in the methods section.

Commonly used optimization algorithms include the root mean square prop (RMSProp), adaptive gradient (AdaGrad), and adaptive moment estimation (Adam) algorithms^31^. Since the Adam algorithm combines the advantages of the RMSProp algorithm and the AdaGrad algorithm, it can be applied to solve a wide range of problems, including models with sparse or noisy gradients. Therefore the optimization algorithm we used Adam's algorithm with the update rule shown in Eq. (3). The parameters $\eta ,\beta_{1} ,\beta_{2} ,\varepsilon$ in which references are taken as 0.001, 0.9, 0.999 and 1e−8, respectively^32^.

3 $$\left\{ \begin{gathered} m_{t} = \beta_{1} m_{t - 1} + (1 - \beta_{1} )g_{t} \hfill \\ v_{t} = \beta_{2} v_{t - 1} + (1 - \beta_{2} )g_{t}^{2} \hfill \\ \theta_{t + 1} = \theta_{t} - \eta \hat{m}_{t} /\sqrt {\hat{v}_{t} + \varepsilon } \hfill \\ \end{gathered} \right.$$

where $m_{t}$ is first moment estimate; $v_{t}$ is second raw moment estimate; $\beta_{1} ,\beta_{2}$ is the decay rate; $\hat{m}_{t}$ is first motion estimation after the gradient modification objectively; $\hat{v}_{t}$ means second raw motion estimation after the gradient modification objectively; $\eta$ is the learning rate; $g_{t}$ is the gradient; $\varepsilon$ is a constant; $\theta_{t}$ is the objective function.

### Method flow

**Figure 3:**
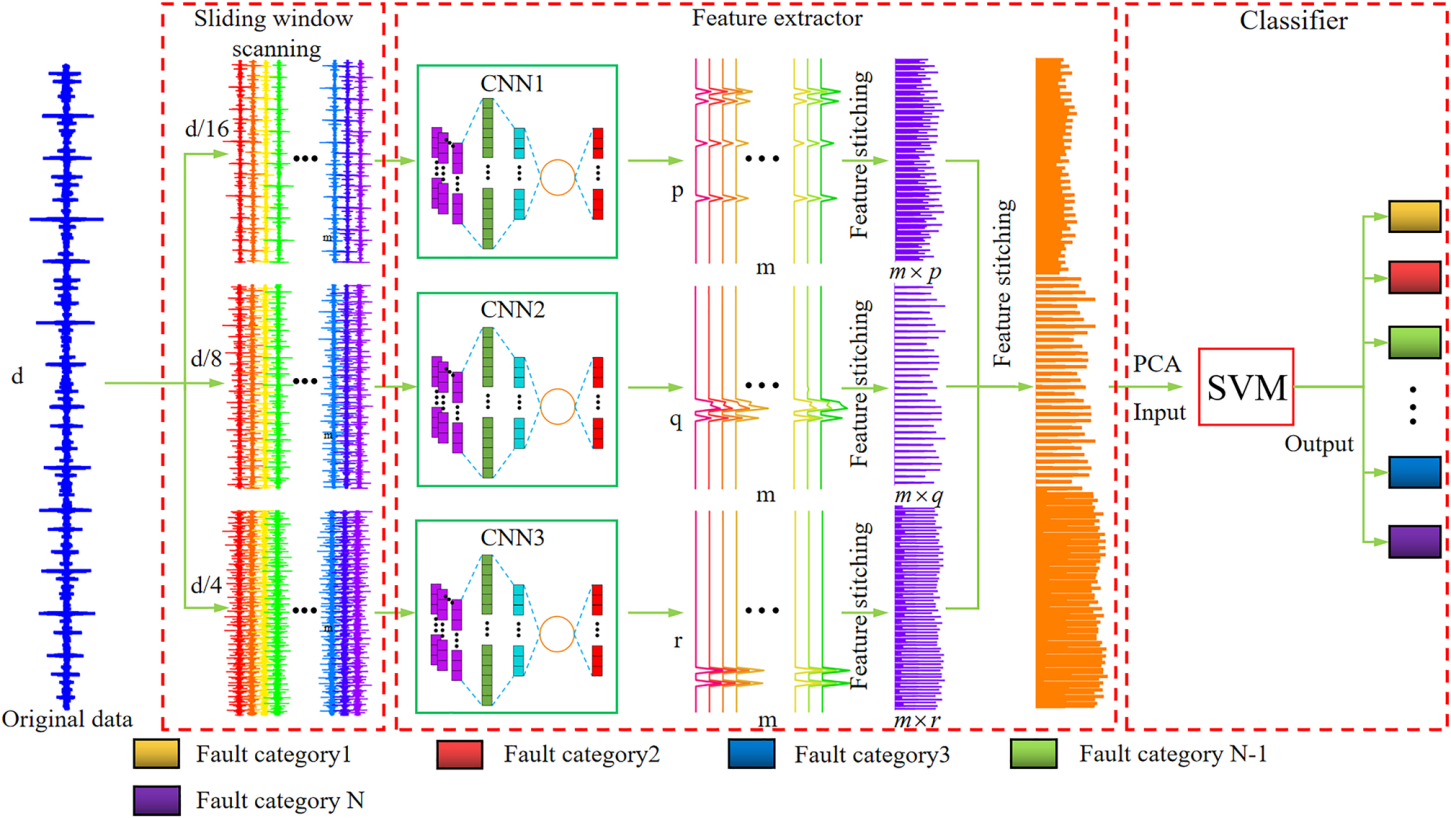
Processing flow.

The process of our mechanical fault diagnosis method is shown in Fig. 3. In Fig. 3, d represents the dimension of the original data, m represents the number of new samples formed from an original sample after sliding window scanning, and p, q, r represent the feature dimensions extracted by CNN respectively. First, the original data is scanned through a sliding window to form sub samples of three scales. Then three sample subsets are input to three CNN feature extractors respectively for feature extraction. The three CNN feature extractors CNN1, CNN2 and CNN3 have the same structure but with different input dimensions. After feature extraction, the feature vectors on three scales of the same sample are spliced for feature reconstruction to form a new feature set with the same number of samples as the original sample set. Assuming that the reconstructed feature dimension is M, the value of M can be calculated by Eq. (4). Finally, the new feature set is input into the support vector machine for fault classification. Support vector machine has unique advantages in dealing with small samples and nonlinear problems, so we use SVM to classify the classifier. Before features are input to SVM, they are dimensionally reduced by PCA. The purpose of this is to remove the miscellaneous information in the data, improve the calculation efficiency, and retain 95% of its information^33^.

4 $$M = m(p + q + r)$$

where M is the dimensionality of the reconstructed features; m is the number of new samples after the sliding window scan; p, q and r are the dimensionality of the features after CNN extraction respectively.

### Experiment

**Figure 4:**
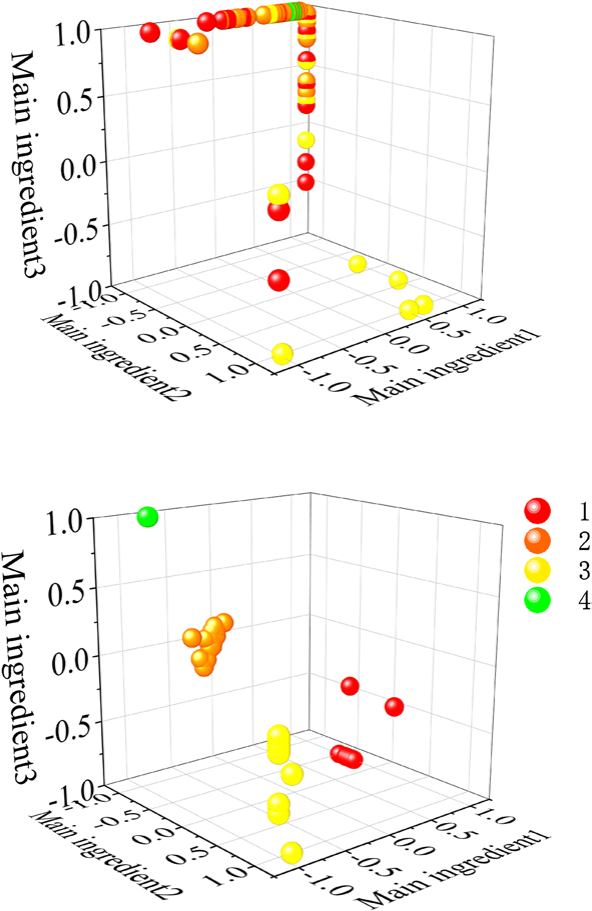
Visualization of bearing fault data of Case Western Reserve University.

First, the training set, validation set and test set are expanded. The data visualization before expansion is shown in Fig. 4a. One sample is expanded to 32 subsamples. Thus, the sample sets of three scales are 2624 × 7538, 2624 × 15,077 and 2624 × 30,154 respectively. Here, 2624 represents the number of samples, and 7538, 15,077 and 30,154 represent the data points of samples respectively. The number of training set, validation set and test set samples in the three sample sets are 1824, 512 and 288 respectively. Use the training set and validation set of the three scale sample sets to CNN1, CNN2 and CNN3 respectively for training to extract the output of the full connection layer as the feature. Use the test set separately after training, and extract the feature. A new sample set of 82 × 6144 is formed after feature splicing and reconstruction. Before the new sample set is input into SVM for classification, PCA dimension reduction processing is carried out first. The reduced dimension data is visualized as shown in Fig. 4b (Take the first three dimensions of the reduced dimension data). Main ingredient1, Main ingredient2 and Main ingredient3 in the figure represent the 1st, 2nd and 3rd principal components respectively after the PCA dimensionality reduction. The partition ratio of training, validation and test sets of the new sample remains same as before.

It can be seen from Fig. 4a that the four types of original fault data are gathered together and entangled with each other. However, it can be seen from Fig. 4b that after the original data has been scanned by the sliding window and processed by the feature extractor, the same data has a high degree of aggregation and the distance between classes is large so that the four types of fault data can be better distinguished. In order to evaluate the quality of the clusters more accurately, we introduce the concept of the average silhouette coefficient^34^. It can be used to describe the degree of denseness and sparseness of classes and is mathematically described as follows:

5 $$s_{i} = \frac{{b_{i} - a_{i} }}{{\max (a_{i} ,b_{i} )}}$$

where $s_{i}$ is the silhouette coefficient of class i; $a_{i}$ is the average distance between the sample point and other sample points of the same class; $b_{i}$ is the average distance between the sample point and sample points of other classes.

The average silhouette coefficient S can be expressed in the following equation.

6 $$S = \frac{1}{n}\sum\limits_{i = 1}^{n} {s_{i} }$$

where n is the number of classes.

It is easy to see that the value of S should be between [− 1, 1]. A larger S means a larger gap between the intra-class distance and the inter-class distance, the better the clustering effect. The average silhouette coefficient of the extracted data was calculated to be S = 0.950. A large number, indicating a good clustering effect.

**Figure 5:**
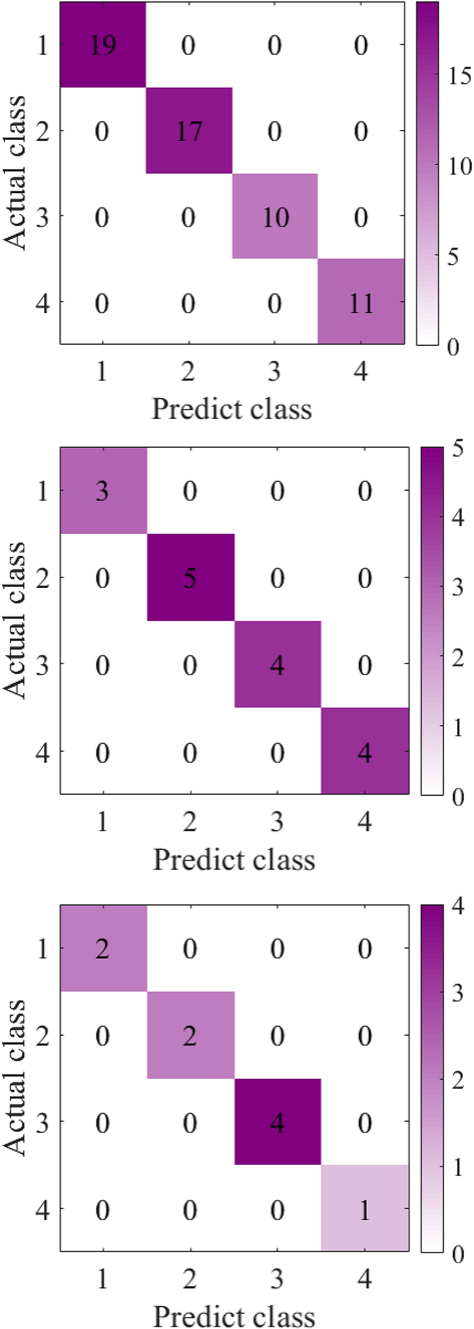
Confusion matrix.

After the feature extracted data is dimensionally reduced by PCA (95% information is retained), the training set is input into SVM for training, the validation set is validated, and the test set is used for model testing. During SVM training, a fivefold cross validation method is adopted and the RBF is selected as the kernel function. In order to obtain the best parameters, the grid search method is used to obtain the values of hyper-parameters C and $\lambda$. The best hyper-parameters obtained are: C = 0.1, $\lambda = 0.72$. The confusion matrices of the training set, validation set and test set are shown in Fig. 5a,b,c respectively. It can be seen from Fig. 5 that the proposed method does not have wrong scores on the training set, validation set and test set, and the four types of fault identification accuracy and total identification accuracy reach 100%.

**Figure 6:**
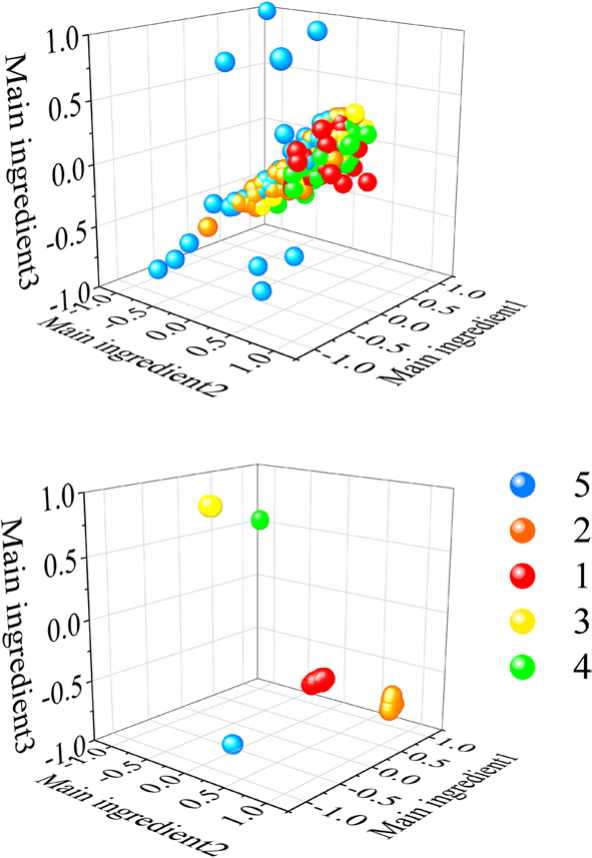
Visualization of bearing fault data of Xi'an Jiaotong University.

**Figure 7:**
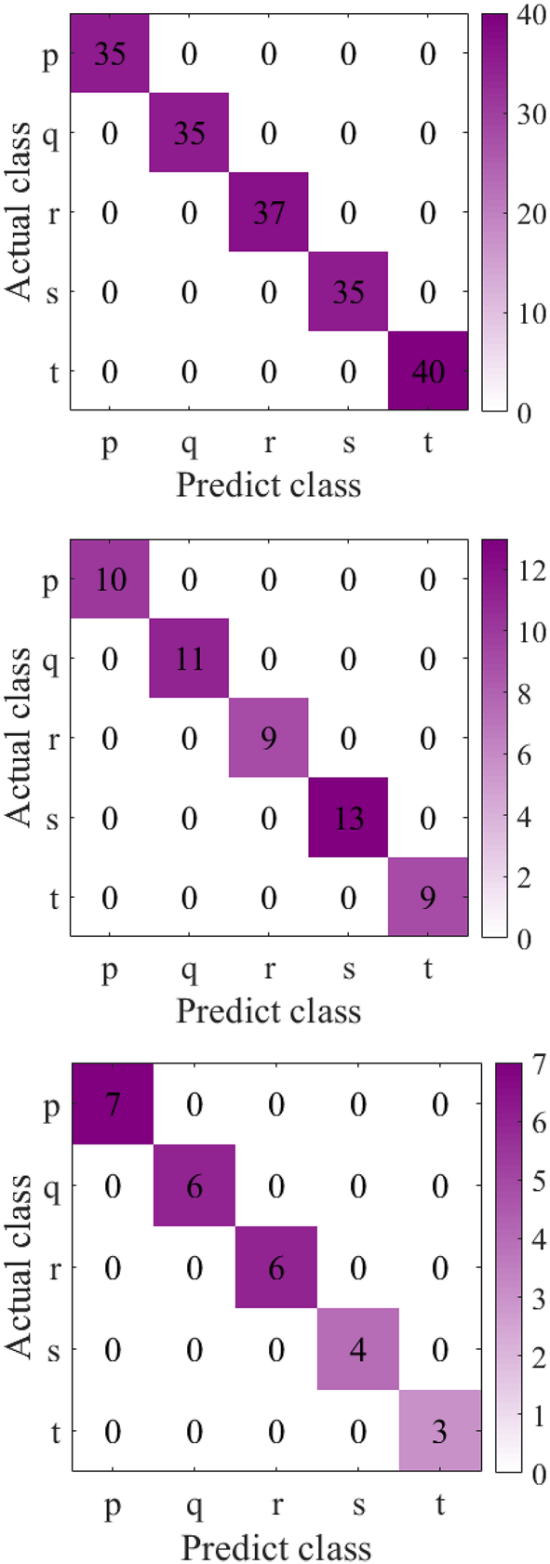
Bearing fault confusion matrix of Xi'an Jiaotong University.

**Figure 8:**
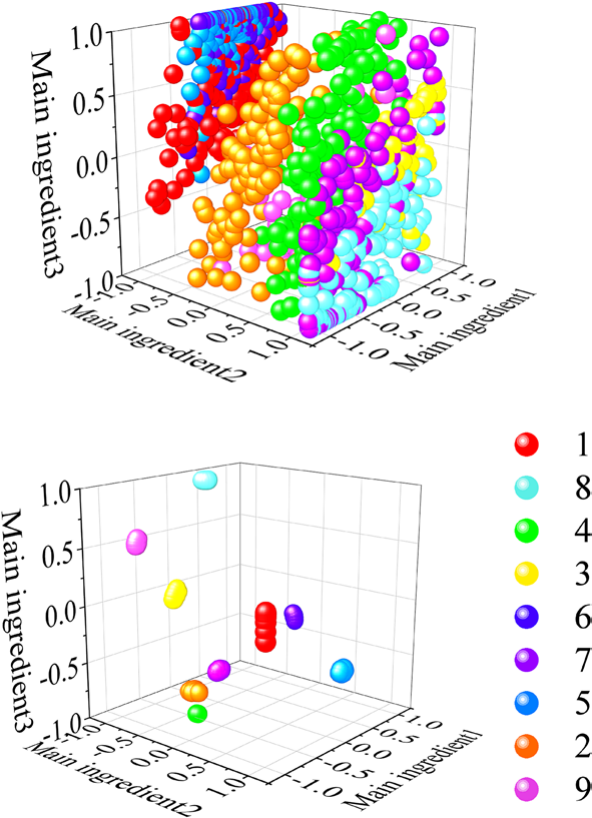
University of Connecticu gear failure data visualization.

**Figure 9:**
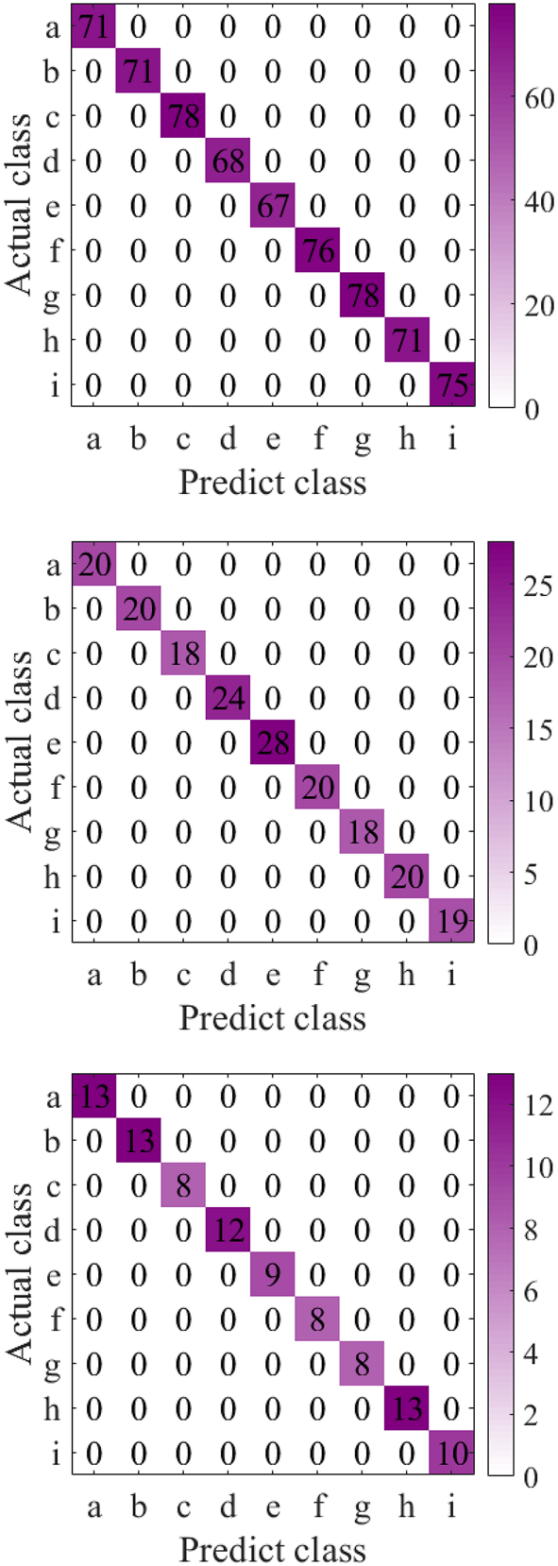
University of Connecticu gear fault confusion matrix.

To verify the generalization performance of the proposed method, we conducted experiments on the bearing fault dataset of Xi'an Jiaotong University and the University of Connecticu gear fault dataset. The data visualization and confusion matrix are shown in Figs. 6, 7, 8 and 9 respectively. The average silhouette coefficient were 0.981 and 0.974 respectively.

## Discuss

### Comparison of recognition accuracy

**Figure 10:**
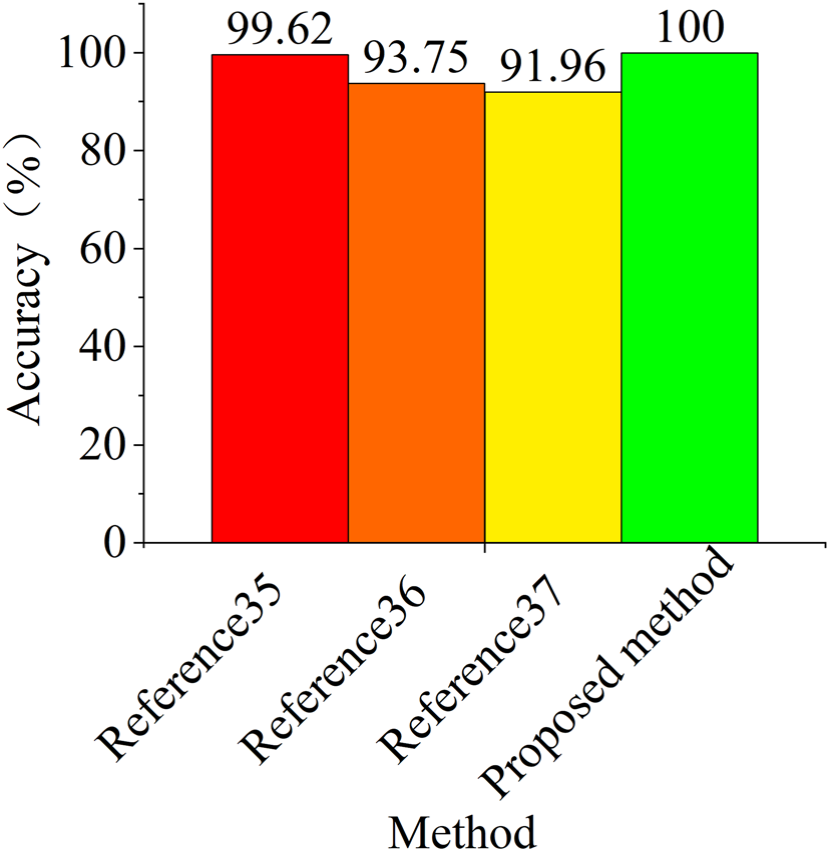
Precision comparison.

Case Western Reserve University bearing fault data set is a relatively famous data set, which has been studied by many scholars. Among them, there are not only traditional fault diagnosis methods based on professional experience for feature extraction, but also intelligent diagnosis methods that are more popular recently. Literature 35 and 36 use traditional methods for fault diagnosis, while literature 37 uses intelligent methods for fault diagnosis^35–37^. In order to reflect the superiority of the method proposed in this paper in recognition accuracy, we compare the total recognition accuracy of the method proposed in this paper with that of literature 35, 36 and 37 on the test set. The comparison results are shown in Fig. 10. The precision of literature 35, literature 36, literature 37 and this method is 99.62%, 93.75%, 91.96% and 100% respectively (Five experiments were carried out and averaged). The identification accuracy of the method in this paper is 0.48% higher than that of reference 35, and more than 6% higher than that of the other two literature methods. It can be seen that the method in this paper has certain advantages over the other three methods.

In order to further reflect the advantages of the proposed method, a comparison test was conducted between the proposed method and other conventional methods. The experimental data are still used from the well-known Case Western Reserve University bearing fault dataset, and the details of the data are shown in Table 1 for the statistical properties of the Case Western Reserve University bearing fault dataset. We conducted comparative experiments using Support Vector Machines (SVM), Long Short-Term Memory (LMST), One-Dimensional Convolutional Neural Networks (1D-CNN) and Extreme Gradient Boosting (XGBoost) respectively. To make the results more convincing, we made the structure of the 1D-CNN consistent with that of the feature extractor. The parameters of the SVM were kept consistent with the classifier. The LMST used two LMST layers and one fully-connected layer, with the fully-connected layer as the output layer. The average recognition accuracy on the test set is shown in Table 2.

**Table 2:** Comparison of the accuracy of the five methods.

Method	Average recognition accuracy (%)
SVM	22.2
XGBoost	33.33
1D-CNN	42.22
LSTM	22.22
Proposed method	100

It can be seen from Table 2 that except for the methods we proposed, the identification accuracy of other methods is not very high. The highest recognition accuracy among the remaining four methods was 42.22% and the lowest was 22.22%. Among them, SVM and XGBoost are both traditional machine learning methods. Although they have certain advantages in processing small sample data, their feature extraction ability is weak, resulting in low recognition accuracy, which often requires manual extraction of features before using them for classification and recognition. In contrast, 1D-CNN and LSTM belong to deep learning methods. Although they have strong feature extraction capability, their low recognition accuracy is due to the small sample size and the under-learning of model parameters.

Our proposed method, however, combines the advantages of SVM and 1D-CNN, resulting in a high recognition accuracy. Although it may seem that both SVM and 1D-CNN are traditional methods. However, by designing a unique network structure, we enable the feature extractor to extract features from a subset of samples at three scales. Although the sample set after feature reconstruction has the same number of samples as the original dataset, the new sample set does contain more useful features. Therefore, when the SVM is used again for recognition, 100% recognition accuracy can be achieved. This further demonstrates that our proposed method can effectively handle bearing fault data with small samples.

### Generalize performance

It can be seen from Fig. 6a that five types of fault data are gathered together before feature extraction, and five types of data are intertwined with each other. It can be seen from Fig. 6b that after feature extraction, the fault data of the same kind gather together, and there is a large distance between classes. It can also be seen from 8 that after feature extraction, 9 types of fault data are obviously separated. Figure 7 shows the confusion matrix of bearing faults in Xi'an Jiaotong University. The five types of faults are correctly classified in the training set, verification set and test set. Figure 9 shows the confusion matrix of the University of Connecticu gear fault. It can be seen that nine types of faults are also correctly classified in the training set, verification set and test set. Our method has a good effect on the bearing fault data set of Xi'an Jiaotong University and the gear fault data set of the University of Connecticu. The precision of each type of fault and the total precision has reached 100%. The proposed method not only has a good recognition effect on the new bearing data set but also performs well on the gear data set. This proves the effectiveness and strong generalization ability of our method.

## Method

### One dimensional convolutional network

Convolutional neural networks are usually used to process two-dimensional data such as images. However, in order to adapt to the processing of one-dimensional data such as voice and text, many scholars have proposed a one-dimensional convolutional neural network (1D-CNN) model^38^. 1D-CNN is similar to CNN in structure, including input layer, convolution layer, pooling layer, full connection layer and output layer. The difference is that the convolution layer in 1D-CNN uses a one-dimensional convolution kernel. If the jth convolution feature map of the h convolution layer is x$x_{j}^{h}$, then:

7 $$x_{j}^{h} = f\left( {\sum\limits_{i = 1}^{{N_{G} }} {x_{i}^{h - 1} } \otimes k_{ij}^{h} + b_{j}^{h} } \right)$$

where $f( \bullet )$ is the activation function; $\otimes$ is convolution operation; $N_{G}$ is the number of input characteristic graphs of the h layer convolution layer; $k_{ij}^{h}$ is the convolution kernel corresponding to the ith input feature and the jth output feature of the h convolution layer; $b_{j}^{h}$ is the offset term.

Loss function. The loss function in the feature extractor adopts the cross entropy loss function, which is given by the following formula:

8 $$Loos = - \sum\limits_{i = 1}^{\begin{subarray}{l} Output \\ size \end{subarray} } {y_{i} } \log \hat{y}_{i}$$

where $y_{i}^{{}}$ is the true label of the ith sample; $\hat{y}_{i}^{{}}$ is the prediction label of the ith sample.

### Support vector machines

The support vector machine theory was first proposed by Vapnik^39^ when dealing with small sample data sets. Its main idea is to construct a hyperplane to maximize the distance between the sample set and the hyperplane, which can finally be transformed into a quadratic programming problem as shown in Formula (9).

9 $$\left\{ \begin{gathered} \mathop {\min }\limits_{\omega ,b} \frac{1}{2}\left\| \omega \right\|^{2} + C\sum\limits_{i = 1}^{n} {\xi_{i} } \hfill \\ s.t.y_{i} (\omega x_{i} + b) \ge 1 - \xi_{i} \hfill \\ \end{gathered} \right.$$

where $\omega$ is the weight; b is the offset term; $\xi$ is relaxation factor; C is the penalty factor.

When Lagrange multiplier and duality are introduced, the decision function becomes the following formula:

10 $$f(x) = {\text{sgn}} \left( {\sum\limits_{i = 1}^{n} {\alpha_{i} y_{i} } K(x_{i} ,x) + b} \right)$$

where $K(x_{i} ,x)$ is the kernel function. Because the Gaussian radial basis function has excellent nonlinear mapping ability, we choose the Gaussian radial basis function kernel function.

11 $$K(x_{i} ,x) = \exp \left( {\frac{{ - \left| {x - x_{i} } \right|^{2} }}{{g^{2} }}} \right)$$

where g is the kernel parameter.

## Conclusions

To deal with the two challenges of difficult feature extraction and sample size of faulty datasets, we propose the method in this paper. The samples are expanded by sliding window scanning to compensate for the small sample size that cannot meet the deep learning training requirements. Automatic extraction of fault features is achieved by using three CNNs with the same structure to form a feature extractor. Feature reconstruction is achieved by feature stitching and the reconstructed features are fed into the SVM for classification. Experiments were conducted on three datasets. The results show that the proposed method not only easily meets the two challenges, but also has high precision and strong generalisation capability.

However, the experimental dataset on which we conducted our study are the signals collected by the provider after the device has been running smoothly. They are steady-state signals. Further study is needed for non-stationary signals, such as those collected with continuous changes in rotational speed or other non-stationary conditions.

## Data Availability

As the data on the official website of Case Western Reserve University ha-s been removed. Therefore, We have provided a valid github site (https://github.com/yyxyz/CaseWesternReserveUniversityData). The original bearing data set of Xi'an Jiaotong University is available on its official Google drive (https://drive.google.com/open?id=1_ycmG46PARiykt82ShfnFfyQsaXv3_VK). The original gear data set of the University of Connecticut is available on the website (https://figshare.com/articles/Gear_Fault_Data/6127874/1).
